# Progress in Targeted Alpha-Particle Therapy. What We Learned about Recoils Release from *In Vivo* Generators

**DOI:** 10.3390/molecules23030581

**Published:** 2018-03-05

**Authors:** Ján Kozempel, Olga Mokhodoeva, Martin Vlk

**Affiliations:** 1 Czech Technical University in Prague, Faculty of Nuclear Sciences and Physical Engineering, Prague CZ-11519, Czech Republic; martin.vlk@fjfi.cvut.cz; 2 Vernadsky Institute of Geochemistry and Analytical Chemistry, Moscow 119991, Russia; olga.mokhodoeva@mail.ru

**Keywords:** targeted alpha therapy, nuclear recoil, *in vivo* generators, radium, ^223^Ra, actinium, astatine, bismuth, alpha particle, decay

## Abstract

This review summarizes recent progress and developments as well as the most important pitfalls in targeted alpha-particle therapy, covering single alpha-particle emitters as well as *in vivo* alpha-particle generators. It discusses the production of radionuclides like ^211^At, ^223^Ra, ^225^Ac/^213^Bi, labelling and delivery employing various targeting vectors (small molecules, chelators for alpha-emitting nuclides and their biomolecular targets as well as nanocarriers), general radiopharmaceutical issues, preclinical studies, and clinical trials including the possibilities of therapy prognosis and follow-up imaging. Special attention is given to the nuclear recoil effect and its impacts on the possible use of alpha emitters for cancer treatment, proper dose estimation, and labelling chemistry. The most recent and important achievements in the development of alpha emitters carrying vectors for preclinical and clinical use are highlighted along with an outlook for future developments.

## 1. Introduction

Targeted alpha-particle therapy (TAT) is the most rapidly developing field in nuclear medicine and radiopharmacy. The basic advantage of TAT over commonly used β^−^ emitting radionuclides therapy lies in the irradiation of fewer cancer cells, micrometastases or tumors by an emission of a single alpha particle or by a cascade of heavy alpha particles from close vicinity. The 2^+^ charged α particles with high linear-energy transfer (LET) lose the maximum of their energy close to the Bragg peak at the end of their track. The range in tissues is about 50–100 µm depending on the alpha-particle energy. The energy deposition then occurs in a very small tissue volume and with high relative biological effectiveness (RBE) [1]. This is fully true for single α particle decays. However, so called *in vivo* generators [2] that provide, typically, four α decays, depending on the selected radionuclide system, suffer from the nuclear recoil effect, causing at least partial release of daughter radioactive nuclei from the targeting molecule or a delivery vehicle. In such cases, an unwanted radioactive burden is spread over the body and its elimination is limited [3].

Even though recent developments brought significant clinical results [4,5] and novel insights into the problem of the nuclear recoil effect were gained [6,7,8,9], neither a detailed analysis nor exhaustive discussion has been undertaken to solve this problem. Furthermore, proper targeting and dosimetry on a subcellular level has become crucial, and advantageous use of theranostic isotopes or theranostic isotope pairs is becoming very important in therapy prognosis [10].

The nuclear recoil effect causes the release of radioactive daughter nuclei from the original radiopharmaceutical preparations. It may lead to unwanted irradiation of healthy tissues that may cause severe radiotoxic effects like organ dysfunction (e.g., kidneys), secondary tumorigenesis, etc. [11]. The released activity and radioactive daughter nuclei fraction as well as their metabolic fate, therefore, need to be estimated and carefully evaluated. Additionally, the key *in vivo* parameters of the radiopharmaceuticals for TAT like e.g.,: biological half-life, carrier *in vivo* stability, uptake in the reticulo-endothelial system (RES), plasma clearance, elimination routes, etc. may play an important role.

Dosimetric studies should separately evaluate in detail the contributions of a radiolabelled targeted vector, its labelled metabolites, liberated mother nuclide as well as daughter recoils. The evaluation should be performed either experimentally or using mathematical models. Various techniques were used for *ex vivo* evaluation of activity distribution in tissue samples. They include, e.g., an alpha camera [12] or a timepix detector [13] to assess the distribution in sub-organ or cellular levels. Also the possibility of the Cherenkov radiation luminescence imaging technique for α emitters employing the co-emitted β^−^ radiations [14] was reported.

Several different approaches were developed regarding the carriers for TAT. Small molecules, particularly those labelled with single α emitters, brought the advantage of fast kinetics even though their *in vivo* stability was not always good. Additional approaches to mitigate radiotoxic effects were studied, e.g., to protect kidneys [15]. Immunoactive molecules like antibodies, antibody fragments, nanobodies or receptor-specific peptides represent another group of highly selective targeting vectors [16].

A relatively novel concept of at least partially recoil-resistant carriers for TAT was developed. It employs nanoconstructs composed of various nanoparticulate materials [6,8,17] that allow further surface chemistry, including antibody targeting. However, the major disadvantageous property of large molecular vectors, e.g., of TiO_2_ nanoparticles (NPs) [18], is their typical uptake in RES and slower *in vivo* kinetics, e.g., when using antibody without surface detergent modulation [19].

This review tries to cover all aspects of TAT from the research and development of production of alpha emitters and labelling techniques to the preclinical and clinical research and applications of the developed radiopharmaceuticals. In order to estimate the potential risks and benefits of TAT, we survey important features of different stages of radiopharmaceutical preparation and the directions of required investigation and development.

## 2. Production of Alpha Emitters

Production of alpha-particle emitters includes, in general, practically all methods for preparation of radionuclide sources—irradiation with charged particles in accelerators, neutron irradiation in a nuclear reactor, separation from long-lived natural radionuclides and various combinations thereof [20,21,22,23,24,25,26,27,28,29,30,31,32,33,34,35]. The great advantage of nuclides decaying in a series over single alpha particle emitters is not only in the higher energy deposition in target tissue but, thanks to the good nuclear characteristics, also the possibility of construction of a radionuclide generator. Selected characteristics and the main production methods for the most common alpha emitters used in various phases of research and use in nuclear medicine are summarized in Table 1. The challenges encountered in the production of alpha emitters were discussed in a recent review [21]; however, a wider clinical spread of alpha emitters depends more on the end-users’ confidence and better understanding of the TAT concept that should help in overcoming the sometimes negative historical experience (e.g., with ^226^Ra).

## 3. General Radiopharmaceutical Issues

Direct and indirect radiolabeling methods are available for single alpha-particle emitters. Since the nuclear recoil effect does not affect the spread of radioactive burden originating from the recoiling radioactive daughters, particularly the ^211^At, a halogen that uses chemistry similar to iodine is very attractive. Furthermore, the radiometals like ^149^Tb and several latter decay series members like ^213^Bi appear to be very promising. Radionuclides decaying by a series of several α decays release radioactive daughter nuclei from the radiopharmaceutical preparations due to the nuclear recoil effect. This effect complicates the labelling strategies and successful dose targeting. In its presence, both the pharmacokinetic properties of the radiopharmaceuticals and the strategies for elimination of the released radioactive burden need to be optimized.

### 3.1. Nuclear Recoil Effect and the Release of Daughter Nuclei

**Figure 1:**
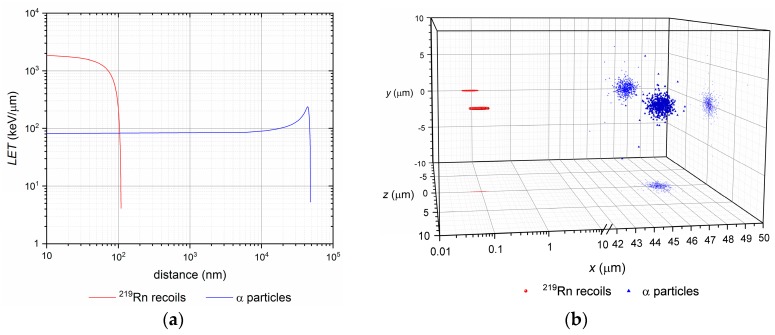
(**a**) Log/log plot of linear-energy transfer (LET) of α particles and ^219^Rn ions vs. their path (distance) in water up to the rest; (**b**) Semi-log 3D plot of final at rest positions of α particles and ^219^Rn ions with their *xy*, *xz* and *yz* plane projections. The recoil, in fact, travels in opposite direction to the emitted α particle (common decay-event origin at *x*,*y*,*z* = 0,0,0).

Due to the momentum conservation law, part of the decay energy is transferred to a daughter nucleus. An approximate value of this energy can be calculated by the mathematical relation:

(1) $$E_{r}=\frac{m_{\alpha }}{M_{r}}Q$$

where *E_r_* is the recoil energy, *m*_α_ the rest mass of an α particle, *M_r_* mass of the recoil and *Q* is the decay energy. The energy distribution ratio between the alpha particle and the recoiling atom is typically 98% to 2%. The amount of energy that the recoil atom reaches is some 100 keV and that is not negligible. Such energy is sufficient to break some 10,000 chemical bonds (assuming 10 eV/one bond). An example of such 109 keV recoil is the ^219^Rn with the range of some 88 nm in a water-like environment (e.g., cells or extracellular matrix). The comparison of LET and ion ranges of α particles and ^219^Rn recoils originating from ^223^Ra decay is shown in Figure 1 and the ranges of ^219^Rn recoils in various materials are summarized in Table 2. Simulations were performed using SRIM code [36]. These factors have a direct impact on radiopharmaceutical stability and purity, as well as on dosimetry and daughter recoils’ distribution in tissues, especially when so called *in vivo* generators are employed. In some cases the radioactive recoils are removed from the radiopharmaceutical preparations and their final formulations before use [37,38].

To mitigate the consequences of the nuclear recoil effect in the body, we propose three methods based on the corresponding theorems:

**Theorem** **1.** Recoils spread mitigation by time—the spread of daughter radioactive ions takes time, so their spread in the organism would also depend on their half-life.

**Proof of Theorem** **1.** The blood flow measured in terms of red blood cells velocity in capillaries ranges between about 1–3 mm/s [39]. Taking into account this value as a reference for passive transport of radiopharmaceuticals in extracellular matrix or in a capillary blood stream, one may compare this displacement time and the half-life of the corresponding released daughter nuclide. While only one half of ^219^Rn atoms (T = 3.96 s) decay roughly in 4 s, the number of atoms of further decay series member ^215^Po (T = 1.78 ms) decreases to ^1^/_1000_ of its initial amount in 17.8 ms, and it thus has practically no time to escape or to be translocated. Thus, the selection of nuclides with favorable decay properties determines this approach.

**Theorem** **2.** Recoils spread mitigation by nanoconstruct size/material—daughter-recoiling nuclide consumes some of its energy while getting through the nanoconstruct.

**Proof of Theorem** **2.** Depending on the nanoconstruct design the stopping power of various materials affects the recoils range. The material and size of the nanoconstruct thus determine the energy loss of recoils in nanoconstruct material. Not only the atomic structure but also the molecular structure and chemical-bond environment affect the stopping ability of the nanoconstruct as a whole [36]. Furthermore, the recoil ion range in nanoconstruct material is limited and its energy is significantly decreased. In general, the stopping power increases with various parameters like atomic weight, electronic density, bond structure, etc. The advantage of spherical nanoconstruct geometry in terms of the stopping efficiency of the nanoconstructs is obvious, and the mother nuclide should be preferably placed in the nanoconstruct core. On the other hand, in case of surface-bonded radionuclide, the probability of daughter recoil ion back-implantation into the nanoconstruct is about 50%.

**Theorem** **3.** Recoils spread mitigation by the nanoconstructs number/depot—even though the recoil ion may escape a nanoconstruct, the probability of its back-implantation or its implantation into surrounding nanoconstruct units is relatively high.

**Proof of Theorem** **3.** In cases when time, nanoconstruct material and size are not sufficient to degrade the recoils energy completely, the released ions may be trapped by a depot of surrounding nanoconstructs or even as mentioned in Theorem 2, by the nanoconstruct itself. This proof is also supported by the fact that both surface and intrinsic labelling strategies yielded quite similar data on *in vitro* stabilities results in terms of total released activity [17]. This method is, however, limited to topical applications of radiopharmaceuticals based on larger nanoconstruct aggregates or agglomerates.

### 3.2. Labelling Chemistry

A fundamental concept of small molecule labelling, e.g., the antibody fragments, peptides and also surface-modified nanocarriers, is based on chelators conjugated throughout a spacer with the vector or the nanocarriers themselves. Spacers are aliphatic or aromatic moieties (C4–C10 or longer) able to establish chemical bonds (e.g., amides, esters, etc.) via nucleophilic substitution, amide formation using carboimides (e.g., dicyclohexylcarbodiimide, diisopropylcarbodiimide) or the Schotten–Baumann reaction of acylhalogenides with amines. The “click reactions” of azides with moieties containing triple bonds play the most important role, e.g., the Huisgen’s 1,3-dipolar addition at elevated temperatures resulting in 1,5- or 1,4-isomers, or Cu(I) catalyzed azide-alkine cycloaddition (preferably resulting in 1,4-product). Cycloaddition reactions help to establish a bond between the spacers and targeted moieties very quickly and efficiently.

**Figure 2:**
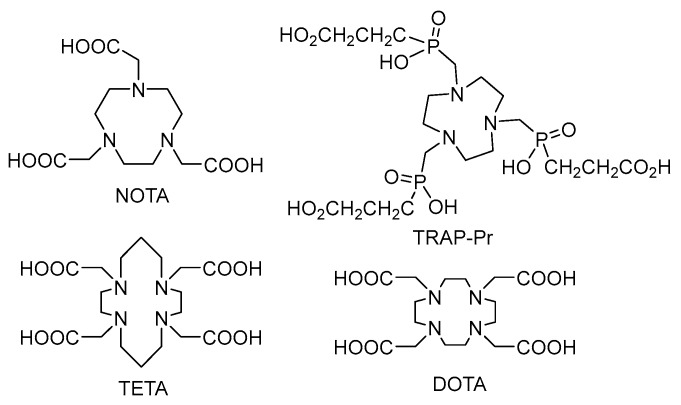
Chemical formulas of cyclic chelators.

Excellent chelators of trivalent metals are the azamacrocyclic ligands based on DOTA, NOTA or TETA analogues (e.g., carboxylic or phosphonic)—see Figure 2. Most of them are commercially available with various spacer lengths and as protected (e.g., *t*-butyl or benzyl) or unprotected derivatives.

These chelators provide very fast trivalent ions complexation kinetics (e.g., Ga, Lu, Tb, Ac, Bi, etc.) depending on pH and temperature. Most of them are used with coordinated stable metals (e.g., Gd) as contrast agents in magnetic resonance imaging (MRI) and they are very often employed as chelators for diagnostic positron emission tomography (PET) radionuclides (e.g., ^68^Ga) as well as beta decaying therapeutical nuclides (e.g., ^177^Lu) [40,41,42,43,44]. During the past few years macrocyclic ligands were also used in TAT as chelators suitable for ^225^Ac or ^213^Bi [44,45]. Thus, DOTA/NOTA like bifunctional chelators are fulfilling the theranostic concept according to which one chelator may be employed for multimodal diagnostic purposes or as α/β^−^ therapeutic agents. Concerning the α emitters, it is interesting that even though the energy released during α decay exceeds several hundred times the Me–C, Me–O or Me–N bond energy (Me—radiometal) and the recoils are released from the carrier, *in vivo* experiments indicate that the use of such delivery systems is also feasible [4,46].

**Figure 3:**
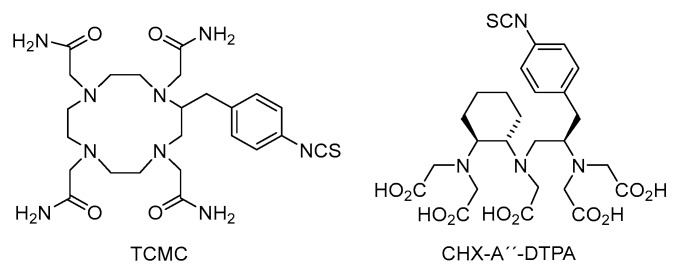
Chemical formulas of TCMC and CHXA”-DTPA chelators.

As already mentioned, labelling procedures proceed quite rapidly, taking dozens of minutes at laboratory or elevated temperatures (up to 95 °C) at pH = 1–5 depending on the central atom and also ligand structure. Several studies indicate that coordination of trivalent gallium by TRAP-Pr at pH = 1–3 and room temperature is more efficient than NOTA, DOTA, TETA analogues under similar conditions. Optimal labelling protocol was established within 10–30 min for ^68^Ga at pH = 3–4 (acetate or citrate buffer) at elevated temperatures (90–95 °C). It was also observed that the presence of trace metal impurities like Zn^2+^, Cu^2+^, Fe^3+^, Al^3+^, Ti^4+^ or Sn^4+^ does not significantly decrease the radiochemical yield while gallium labelling proceeds [47,48]. This ligand is, thus, promising also for other radiometals like ^213^Bi, ^225^Ac. However, under certain conditions macrocyclic ligands form mostly in-cage structures. Depending on the reaction conditions and basicity of the ligands, less thermodynamically stable out-of-cage structures may occur usually when the reaction has been performed at lower temperatures. Employing microwave irradiation may also significantly help to ensure faster formation of in-cage complexes. Experimental ^225^Ac-DOTA-PSMA-617 was synthesized in a microwave reactor at pH = 9 (*TRIS* buffer) within 5 min with radiochemical purity over 98% and specific activity 0.17 MBq/nmol. Similar protocols were employed when synthesized ^213^Bi-DOTATOC and ^213^Bi-Substance P were synthesized, hexadentate DOTA-peptide conjugate being used [49,50]. Both ^213^Bi and ^212^Bi are considered for the purpose. A ^212^Pb-TCMC-trastuzumab conjugate was studied on patients with HER-2 receptor carcinoma and its toxicity, pharmacokinetics and dosimetry were investigated. However, the use of DOTA analogues as chelators of ^225^Ac or ^213^Bi did not solve the toxicity of daughter recoils. A very interesting alternative to the presented α emitters is the ^149^Tb, currently studied in a preclinical immunotherapy. Terbium-149 was separated from isobaric and other impurities including stable zinc by extraction with α-hydroxyisobutiric acid solution (pH = 4) and was directly added to DOTANOC (incubation: 15 min at 95 °C). Subsequent high-performance liquid chromatography (HPLC) confirmed an over 98% purity and high specific activity (5 MBq/nmol) of ^149^Tb-DOTANOC. A similar approach was used for ^149^Tb-DOTA-folate (incubation: 10 min at 95 °C) [51,52]. Labelling of monoclonal antibody CD20 rituximab with ^149^Tb in a mixture of ammonium acetate, ascorbic acid and phosphate-buffered saline (PBS) buffer (pH = 5.5) and 10 min incubation at room temperature resulted in 99% yield and specific activity of 1.11 GBq/mg. Conjugate ^149^Tb-rituximab was prepared using cyclohexane diethylene triamine pentaacetic acid (CHX-A”-DTPA) [53]. This pentaacetic acid analogue is a very interesting ring-opening chelator used in several studies with ^213^Bi-HuM195 on patients with human myeloid leukemia. The TCMC and CHX-A”-DTPA chelators are shown in Figure 3. 

While ^225^Ac, ^213^Bi, ^149^Tb and other radionuclides may be easily coordinated using macrocyclic or DTPA chelating agents, efficient chelator for ^223^Ra, which is currently used in palliative treatment of bone metastasis of prostate cancer, is still not available. Thus, direct sorption of ^223^Ra onto surface or intrinsic labelling of nanocariers, e.g., nanohydroxyapatites, LaPO_4_, SPIONs and others was investigated [6,8,17]. Due to the problematic chemistry of ^211^At several studies were focused on the possibility of trapping astatine into a nanoconstruct (e.g., gold or silver nanoparticles (NPs), ^211^AtCl@US-tubes, TiO_2_), attached to targeting vector via a linker [54,55,56,57]. Synthesized nanoconstructs might be stabilised with polyethyleneoxide or polyethylene glycol (PEG). Retention of the α emitter is also significant in liposomes, where about 81% ^225^Ac retention was observed but the recoil retention was not evaluated [58]. Whereas both the labelling of nanoconstructs or liposomes and stabilization of recoils are quite efficient in comparison with small molecules, the stability of their dispersions (e.g., the hydrodynamic diameter) may significantly vary depending on used material.

### 3.3. Targeting and Clearance

Investigation of how to deliver short-range, high LET radiation to target sites is of key importance. Short α particle range in soft tissues favors their use in the therapy of small lesions, metastases or system-spread diseases like some kinds of leukemia. Depending on the biochemical properties of the radiopharmaceuticals, three targeting strategies could be defined:

1. “self-targeting” based on physiological affinity of the radioisotope to a given tissue; thus radium tends to accumulate in bones or pertechnetate, astatine or iodide in the thyroid;
2. “passive targeting” or “blood circulation and extravasation” is based on accumulation of nanoparticles in the areas around the tumors with leaky vasculature; commonly referred to as the enhanced permeation and retention (EPR) effect [59];
3. “active induced targeting” based on specific ligand-receptor interactions between labelled small molecules, peptides, mAbs and their fragments and target cells; externally activated exposure is also possible (temperature, magnetic field or other activators) [60].

**Figure 4:**
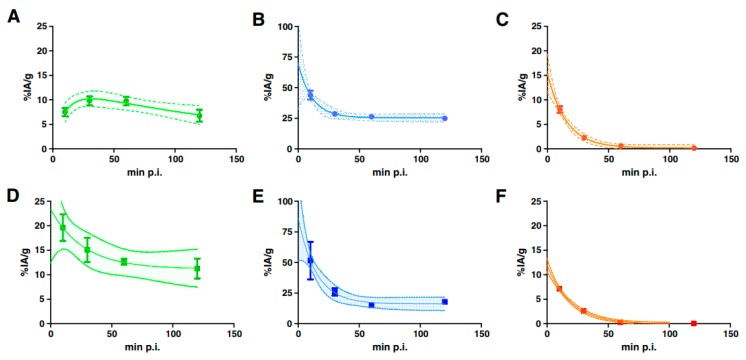
Selected pharmacokinetics of ^213^Bi-DOTATATE in H69 (**A**–**C**) and CA20948 (**D**–**F**) tumor-bearing animals: uptake in tumors (**A**,**D**) and kidney (**B**,**E**), and radioactivity in blood (**C**,**F**) [63].

Taking into account the half-lives of the therapeutic nuclides and the recoiling daughters, their circulation time, biodistribution and clearance play a critical role. Matching radionuclide half-lives and pharmacokinetic profiles of the vehicle systems remains a significant criterion [61]. Radionuclides with half-lives long enough to allow differential tumor accumulation and possibly cellular internalization of radiolabeled molecules have some advantages in therapeutic application, but their toxicity for non-targeted sites should be minimized. The features of recoils’ distribution in the body was discussed by de Kruijff et al. [62]. Pharmacokinetics of the injected radiopharmaceutical could be a function of both time and tumor size. As an example, the data of a preclinical study with ^213^Bi-DOTATATE in animals bearing small and large tumors (50 and 200 mm^3^) using two tumor models: H69 (human small cell lung carcinoma) and CA20948 (rat pancreatic tumor) are demonstrated in Figure 4 [63].

Different approaches have been explored to inhibit the accumulation of both parent and daughter radionuclides in critical organs or acceleration of their clearance: co-injection of lysine with ^213^Bi-labelled conjugate can reduce kidney uptake of ^213^Bi [64], bismuth citrate pre-treatment blocks renal retention of ^213^Bi [65], and oral administration of BaSO_4_ known as a coprecipitating agent of radium reduces the ^223^Ra accumulation in the large intestine [66]. In some cases only locoregional therapy (not intravenous injection) is suited because of the large size or high hydrophilicity of the delivery agent, e.g., encapsulated liposomes or multi-layered nanoconstructs [67]. Imaging methods with the potential for *in vivo* evaluation of the pharmacokinetics of the radionuclides, such as single-photon emission computed tomography (SPECT)/PET/CT imaging are of great importance for assessing the outcome of the therapy.

### 3.4. Dosimetry 

The absorbed dose is defined as an energy delivered to a unit of mass (see Equation (2)).

(2) $$D=\frac{E_{x\;}\left[J\right]}{m_{irr.}\;\;\left[\mathrm{kg}\right]}\;\left[\mathrm{Gy}\right]$$

where the dose, *D* is defined as a ratio of the energy *E_x_* deposited by the radiation passage to the matter in a unit of mass *m_irr_*. This definition is however quite general and does not reflect the specific situation when α emitters and chain decays are used in TAT. This requires precise and accurate dose estimation on all levels, starting from whole body biodistribution down to subcellular level. The example of ^223^Ra decay that produces one α particle and recoiling ^219^Rn ion gives a clear picture of such situation. Let us assume that the cell density equals 1 g/cm^3^, the mass *m_irr._* taken into dose calculation is expressed as the mass of a sphere with the diameter of the ^219^Rn recoil path, and the energy *E_x_* equals the recoil total energy deposition (109.5 keV). In the case of α particle, a sphere with the diameter of 20 µm (single cell dimension) and only partial energy deposition calculated on the basis of LET is considered. Thus the absorbed dose *D* delivered by the ^219^Rn recoil corresponds to 40 kGy in such small volume (total deposited energy of 109.5 keV) while for the α particle it amounts only to 70 mGy over its single-cell path (though the total energy deposited by an alpha particle is 1.83 MeV). To compare the dose in the same mass (or volume), e.g., of one cell, the ratio of the doses delivered by a single α particle and ^219^Rn recoil turns then to 70 mGy to 4 mGy, respectively. Thus the implications for radionuclide targeting on the subcellular level (e.g., internalization into the nucleus or destruction of cell organelles) play an important role and the contribution of recoil ions should not be neglected. In general, the dosimetry should be evaluated separately in the following levels.

#### 3.4.1. Body Level

*In vivo* whole body scans with α emitters may provide very helpful and quite detailed information on the pharmacokinetic and pharmacodynamic properties of radiopharmaceuticals [68,69]. Organ intake values, renal clearance or fecal excretion may be evaluated in this way and the recoil release could be possibly visualized by employing the multiple energetic windows data analysis.

#### 3.4.2. Organ and Sub-Organ Levels

The *ex vivo* sample measurements in animal models and also the *in vivo* imaging can provide overall information on the biodistribution and organ uptake of radiopharmaceuticals [12,70]. Sub-organ distribution may also be visualized and more detailed information on target organ uptake compartments may be gained. Such information is again very important for the estimation of tumor therapy prognosis since some tumors do not express their specific antigens or do not accumulate the targeting vectors in their whole volume.

#### 3.4.3. Cellular and Subcellular Level

Dosimetry on a cellular level should clarify the cell-death mechanisms induced by radiation and damage of cellular compartments including DNA damage. Direct (e.g., DNA double strand breaks) and indirect damage mechanisms (e.g., reactive oxygen species generation) should be considered and further analysis is needed, taking into account also the recoil effects. The standard condition of the radionuclide internalization in the cell need not be necessary. The dose distribution on a subcellular level differs significantly for α particles and for the recoil ion—see Section 3.1. The studies published so far did not evaluate the complete decay and the energy distribution in decay products even though microautoradiographic techniques, in a combination with immuno-staining methods, are available [71]. Single α particle-induced damage visualized in real time was also reported [72] and the stochastic simulation of ^223^Ra α particle irradiation effects on subcellular level was recently performed [73]; however, recoils were not taken into account. The cell-to-cell fluctuations in dose deposition ranged up to about 40%. Interesting results were reported in [74]. In a simplified cellular model, the average number of hits by α particles resulting in a 90% probability of killing exactly one cell was estimated to range from 3.5 to 17.6. However, a better understanding of α particles and the damage induced by the hot recoil atoms is needed to achieve precise proper dose estimation.

Contrary to the efforts of trapping the recoils, an innovative approach that is actually based on the controlled release of recoiling atoms with radioactive nuclei was developed. A novel concept of diffusing α emitter radiation therapy (DaRT) was proposed as a new form of brachytherapy. To treat solid tumors, the method uses α particles employing implantable ^224^Ra-loaded wire sources that continually release short-lived α particle emitting recoils that spread over a few millimeters inside the tumor [75]. Immunogenic cell death seems to significantly influence the overall effect of the therapy. 

## 4. Vectors for Targeted Alpha Particle Therapy

Efficient and specifically targeted carriers need to be developed in order to realize the potential and favorable properties of α emitters. A variety of conventional and novel drug-delivery systems have been investigated for these purposes: biological macromolecules (antibodies, antibody fragments), small molecule compounds (peptides, affibodies) and nanocarriers/nanoconstructs.

### 4.1. Small Molecules

#### 4.1.1. MABG 

[^211^At]-*meta*-astatobenzylguanidine (^211^At-MABG) was synthesized to improve the therapeutic effect for the treatment of malignant pheochromocytoma (PCC) and other diseases [76]. Compared with ^131^I-MIBG, sufficient cellular uptake and suppression of tumor size after single administration of ^211^At-MABG (555 kBq/head) have been reported [77]. A kit method for the high-level synthesis of ^211^At-MABG was also developed [78].

#### 4.1.2. Prostate-Specific Membrane Antigen (PSMA)

**Figure 5:**
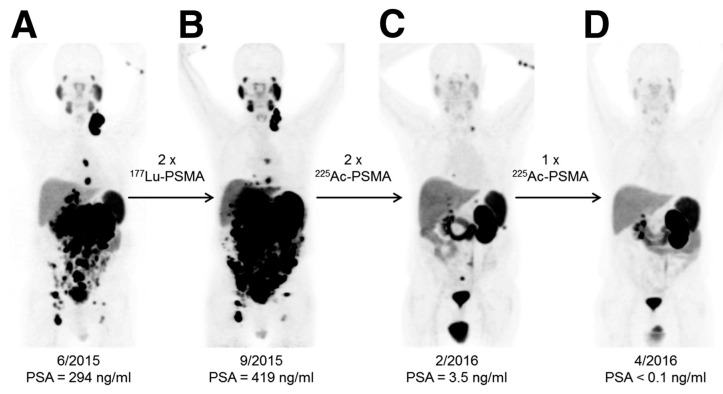
^68^Ga-PSMA-11 positron emission tomography (PET)/computed tomography (CT) scans of a patient comparing the initial tumor spread (**A**); restaging after 2 cycles of β^−^ emitting ^177^Lu-PSMA-617 reveals progression (**B**). In contrast, restaging after second (**C**) and third (**D**) cycles of α emitting ^225^Ac-PSMA-617 shows impressive response. This research was originally published in JNM. Kratochwil et al. ^225^Ac-PSMA-617 for PSMA-Targeted α-Radiation Therapy of Metastatic Castration-Resistant Prostate Cancer**.** *J. Nucl. Med.* 2016, *57*(12), 1941–1944. © by the Society of Nuclear Medicine and Molecular Imaging, Inc. [4].

Clinical salvage therapy with ^225^Ac-PSMA-617 was introduced for patients with advanced mCRPC in whom approved therapies had been ineffective. PSMA (prostate-specific membrane antigen) is a 750 amino acid type II transmembrane glycoprotein; after binding at the tumor cell surface the PSMA ligands are internalized allowing radioisotopes to be concentrated within the cell. A standard treatment activity of 100 kBq/kg administered every 8 weeks presents remarkable anti-tumor activity along with tolerable bystander effects and moderate hematological toxicity [4,5]. Figure 5 shows a patient case with impressive results of TAT in comparison with non-effective ^177^Lu therapy. It was also shown, that ^213^Bi labelled *PSMA* targeting agents induce DNA double-strand breaks in prostate cancer xenografts [79].

#### 4.1.3. Substance P

Clinical experience with the use of peptide carrier Substance P in TAT has recently been reported [80,81]. Patients with recurrent glioblastoma multiforme were treated with 1–7 doses of approx. 2 GBq ^213^Bi-DOTA-Substance P or 1–4 doses of 10 MBq ^225^Ac-DOTAGA-Substance P at two-month intervals. Favorable toxicity profile and prolonged median survival compared to standard therapy were observed.

### 4.2. Biomolecules—Antibodies

A detailed description of mAbs radiolabeling with α emitters has been recently given elsewhere [82,83]. Here we mention only some of the clinical and preclinical studies.

Actimab-A, which represents ^225^Ac conjugated to lintuzumab (anti-CD33 mAb), demonstrated safety and efficacy against acute myeloid leukemia (AML) in two phase 1 trials. Total administered activities ranged from 37–148 kBq/kg and it was found that baseline peripheral blast count is a highly significant predictor of objective response [84]. The phase 2 trial is currently active at 16 clinical trial sites with patients with AML, age 60 and older, who are ineligible for standard induction chemotherapy [85].

^213^Bi-anti-EGFR-mAb radioimmunoconjugate was prepared by coupling ^213^Bi and cetuximab via the chelating agent CHX-A”-DTPA. Intravesical instillation of 366–821 MBq of the ^213^Bi-anti-EGFR-mAb in 40 mL of PBS was applied in recurrent bladder cancer patients revealing well-tolerated therapeutic efficacy [86].

The first-in-human clinical studies of ^212^Pb-AR-RMX (AlphaMedix^TM^, Houston, TX, USA) for therapy of neuroendocrine tumors were announced to have begun. The biodistribution and safety of this peptide derivative vehicle targeting SSTR2-(+) neuroendocrine cancer cells were clinically evaluated using ^203^Pb-AR-RMX. No acute or delayed hematological or renal toxicity was observed [87,88].

Preclinical trials of ^225^Ac-DOTA-anti-PD-L1-BC conjugate have demonstrated promising results in the radioimmunotherapeutic treatment of breast cancer. PD-L1, programmed cell Death Ligand 1, is part of an immune checkpoint system preventing autoimmunity. Anti-PD-L1 antibody (anti-PD-L1-BC) was coupled with p-SCN-Bn-DOTA, and the resulting DOTA-anti-PD-L1-BC conjugate was then labelled with ^225^Ac in sodium acetate. According to the pilot therapeutic studies a single dose of 15 kBq of the ^225^Ac-DOTA-anti-PD-L1-BC (3 mg/kg) increased median survival in a metastatic breast cancer mouse model [12].

8C3 mAb, a 2nd-generation murine antibody to melanin of the IgG isotype, was labelled with ^188^Re or ^213^Bi directly or via CHXA”-DTPA chelator, respectively to prepare a new agent for therapy of metastatic melanoma. There was statistically significant reduction of lesions in the lungs of mice treated with either 400 mCi ^188^Re8C3 or 400 mCi ^213^Bi-8C3 mAb without any undesirable side effects. The unlabeled mAb did not have any effect on the number of the lesions. A statistically significant difference between the ^188^Re and the ^213^Bi treatment was not observed [89].

The efficacy of IgC1k 35A7 mAb (anti-carcinoembryonic antigen, CEA) and trastuzumab (anti-HER2) labelled with ^212^Pb was estimated *in vitro* and *in vivo* in the treatment of small-volume peritoneal carcinomatosis. A strong dose gradient was measured for ^212^Pb-35A7 mAb; it was much more homogeneous for ^212^Pb-trastuzumab. The heterogeneity in mAb distribution was found to be counterbalanced by the presence of bystander effects [90]. Trastuzumab was also labelled with ^225^Ac and studied in a breast cancer spheroids model *in vitro* [91] and with ^211^At in athymic rat model with implanted MCF-7/HER2-18 breast carcinoma cells, in which the median survival almost doubled [92]. 

The small molecule of antibody fragment anti-HER2 2Rs15d Nb was studied as a vehicle of ^225^Ac [93] and ^211^At [94]. The labelling was performed via the bifunctional chelating agent p-SCN-Bn-DOTA for ^225^Ac and three different prosthetic groups m-eATE, SGMAB, MSB for ^211^At using random and site-specific labelling approaches. All prepared conjugates showed efficient degree of internalization in HER2 + SKOV-3 cells justifying their further *in vivo* evaluation.

Poly(ADP-ribose)polymerase-1 (PARP-1), the nuclear protein which exhibits the ability to target directly chromatin, was functionalized with ^211^At for the therapy of high-risk neuroblastoma. The prepared ^211^At-MM4 conjugate demonstrated cytotoxicity to several cell lines [95].

### 4.3. Macromolecules and Nanoconstructs

Conceptual differences in clinical translation of the above vehicles were pointed out. For instance, antibody conjugates target the cell surface and tend to have limited access to solid tumors [96], whereas radiolabeled peptides are more desirable due to straightforward chemical synthesis, versatility, easier radiolabeling, optimum clearance from the circulation, faster penetration and more uniform distribution into tissues, and also lower immunogenicity [97,98]. Nanoparticle-based systems have been designed to improve biodistribution, stability, specificity, pharmacological and targeting properties, daughter retention, as well as to exploit the theranostic approach [99,100,101].

Nanoparticles with two layers of cold LaPO_4_ deposited on the core surface (LaPO_4_ core and core +2 shells) were synthesized and labelled with either ^223^Ra or ^225^Ra/^225^Ac. The NPs were additionally coated with GdPO_4_ and gold shells demonstrating retention of both parents and daughters (over 27–35 days) without diminishing the tumoricidal properties of emitted α particles. Consequent conjugation of LaPO_4_ NPs to 201b mAb, targeting trombomodulin in lung endothelium was carried out using a lipoamide polyethylene glycol (dPEG)-COOH linker. Efficacy of the NPs*-*antibody conjugate system was demonstrated on reduced EMT-6 lung colonies [102,103].

Novel nuclear-recoil-resistant carriers of ^223^Ra based on hydroxyapatite were developed [17,104]. Two strategies were used to prepare the nanoconstructs: the surface and the intrinsic (volume) labelling. High labelling yields as well acceptable *in vitro* and *in vivo* stabilities over the period of ^223^Ra half-life make the developed nanoconstructs promising for targeted cancer therapy, e.g., bone matrix targeting [17]. Similarly, the ^223^Ra labelled CaCO_3_ microparticles were successfully tested in a mice model with ES-2 and SKOV3-luc intraperitoneal ovarian cancer xenografts resulting in considerably reduced tumor volume or a survival benefit [105].

The Au-S-PEG-Substance P (5-11) bioconjugates were proposed to utilize the formation of a strong bond between metallic gold and astatine for binding ^211^At to the biomolecule. Gold NPs were conjugated with Substance P (5-11), neuropeptide fragment with high affinity to neurokinin type 1 receptors on the glioma cells, through HS-PEG-NHS linker. They were then labelled with ^211^At by chemisorption on the gold surface. The radiobioconjugates were stable for 24 h in human serum and cerebrospinal fluid, exhibiting high toxicity to glioma cancer cells. However, only local drug application, not intravenous injection, was recommended because of their relatively large size and high hydrophilicity [57,106].

Substance P (5-11) (SP) was also used for functionalization of nanozeolite-A loaded with ^223^Ra for targeting glioma cancer cells. The small (<5%) release of the daughter radionuclides from the prepared bioconjugate ^223^Ra-A-silane-PEG-SP (5-11) and the ability of zeolite NPs to re-adsorption of recoiled ^223^Ra decay products (as a molecular sieve and as a cation-exchanger) along with high receptor affinity toward NK-1 receptor expressing glioma cells *in vitro* make ^223^Ra-A-silane-PEG-SP (5-11) promising tool for TAT [107]. Nevertheless, like the preceding vehicle it was not recommended for intravenous injection.

Nanocarriers composed of amphiphilic block copolymers, i.e., loaded polymersomes, make it possible to keep the recoiling ^225^Ac daughters and causing complete destruction of spheroidal tumors. Nevertheless, more studies are necessary to evaluate the *in vivo* recoil-retention effectivity [108].

Nanocarriers in the form of lipid vesicles targeted to PSMA were labelled with ^225^Ac and compared with to a PSMA-targeted radiolabeled antibody. It was found that targeted vesicles localize closer to the nucleus while antibodies localize near the plasma membrane. Targeted vesicles cause larger numbers of dsDNA breaks per nucleus of treated cells compared with radiolabeled mAb [109].

Interstitial vehicles in the form of pH-tunable liposomes encapsulating chelated ^225^Ac were designed to enhance the penetration in solid tumors, which is usually limited for radionuclide carriers. The liposomes were composed of 21PC:DSPA:cholesterol(chol):DSPE-PEG:Rhd-lipid. In the slightly acidic tumor interstitium (7.4 > pH > 6.0) a pH-responsive mechanism on the liposome membrane results in the release of the encapsulated radioactivity [110]. This study together with refs. [4,5] actually supports the concept of DaRT therapy [75] in large solid tumors and metastases.

## 5. Summary

Targeted alpha-particle therapy is a very promising and effective therapeutical tool against cancer. This brief overview of recent developments shows great potential in solving partial pitfalls of this method mainly related to the nuclear-recoil effect. We speculate that two major strategies in TAT field are very likely to develop further—firstly, the use of single α particle emitters and/or carriers able to stop the spread of recoils labelled with chain α emitters; and, secondly, the use of carriers providing controlled release of chain α particle emitters (DaRT concept). While the former field would just apply already-known facts, the latter brings a relatively new concept in the TAT, with an overlap to immunologic signaling and cell death. Despite the many uncertainties and problems in TAT, e.g., concerning the proper dose targeting, it should be pointed out that successful treatment cases in animal models have already been reported for both strategies. Also, recent clinical trials showed that patient benefits prevailed over potential risks. Further research is, however, needed to clarify the dosimetry on all levels and to eliminate the unwanted spread of radioactive burden over the body and the induction of secondary malignancies. TAT should, therefore, become additional and equivalent tools in truly personalized medicine.

## Figures and Tables

**Table 1:** Summary and properties of the most relevant alpha particle emitters suitable for nuclear medicine applications.

Radionuclide System *	Half-Life	*E*_α*max*_/*E_chain_* [MeV]	Production	Status	References
^149^Tb	4.12 h	3.97	^152^Gd(p, 4n)^149^Tb	Research	[22,23]
^211^At	7.2 h	5.87	^209^Bi(α, 3n)^211^At	Clinical trials	[24,25]
^229^Th/	7340 years	5.83/27.62	^229^Th decay	Clinical trials	[26,27,28]
^225^Ac//	10 days		^226^Ra(p, 2n)^225^Ac		
^213^Bi	46 min		^232^Th(p, x)^225^Ac		
^227^Ac/	27 years	5.87/26.70	^227^Ac/^227^Th/^223^Ra decay	Clinical praxis	[29,30,31,32]
^227^Th/	18 days				
^223^Ra	11 days				
^228^Th/	1.9 years	5.69/27.54	^228^Th/^224^Ra decay	Formerly in c.p., Research	[33,34,35]
^224^Ra/	3.7 days				
^212^Pb	10.6 h				

* Note that in the case of chain decaying nuclides, not all members of the decay chain are included. Further characteristics of the mainly used chain nuclide are provided in bold.

**Table 2:** Ranges of 109 keV ^219^Rn ions in selected materials.

Material	Range (nm)
Au	11
ZrO_2_ ICRU-712	26
Al_2_O_3_ ICRU-106	27
TiO_2_ ICRU-652	28
SiO_2_ ICRU-245	46
adult cortical bone	53
human blood	85
prostate tissue	87
water	89
nitrogen gas	76,000
